# Biomarkers to enhance accuracy and precision of prediction of short-term and long-term outcome after spontaneous intracerebral haemorrhage: a study protocol for a prospective cohort study

**DOI:** 10.1186/s12883-015-0384-3

**Published:** 2015-08-12

**Authors:** A. Kumar, P. Kumar, S. Misra, R. Sagar, P. Kathuria, D. Vibha, S. Vivekanandhan, A. Garg, B. Kaul, S. Raghvan, S. P. Gorthi, S. Dabla, C. S. Aggarwal, Kameshwar Prasad

**Affiliations:** Department of Neurology, Neurosciences Centre, All India Institute of Medical Sciences, New Delhi, India; Department of Neurology, Safdarjung, Hospital, New Delhi, India; Department of Neurology, Research and Referral Army Hospital, New Delhi, India; Pt. B.D. Sharma, PGIMS, Rohtak, India; Sir Ganga Ram Hospital, New Delhi, India

**Keywords:** Biomarkers, Intracerebral hemorrhage, Prediction, Outcome, Multicentric study

## Abstract

**Background:**

Several studies reported prognostic value of biomarker in intracerebral hemorrhagic (ICH) but they are either preliminary observation or inadequately powered to analyse independent contribution of biomarkers over and above clinical and neuroimaging data.

**Objective:**

To examine whether the biomarker can significantly add to the predictive accuracy of prognosis of ICH.

**Method/design:**

In a multi-centric prospective cohort study, 1020 patients with ICH within 72 hours of onset are being recruited. After obtaining written informed consent from patients/proxy, venous blood sample (10 ml) is being collected and analysed for C-reactive protein (CRP) level, S100B, Glial fibrillary acidic protein (GFAP), Troponin, change in leukocyte count and Copeptin levels. The patients are telephonically followed using stroke scales (Barthel Index and modified Rankin Scale) at 3, 6, 12 months and 2 years after the recruitment.

**Discussion:**

This protocol will aim at predicting the short term or long term prognosis with the use of clinical, neuroimaging and biomarkers in order to help clinician to stratify patients for early referral or intervention.

## Background

Stroke has emerged as the second most common cause of mortality worldwide and a major public health problem. Intracerebral haemorrhage (ICH) accounts for approximately 20-30 % of acute strokes in India and is still associated with a mortality of up to 35–50 % [23]. There are several fold higher incidence rates of ICH (61/100,000) in Asian countries [11], including India than in western countries. It is more common in men than in women [14]. Overall, the prognosis for ICH is poor: 37-47 % of patients die within the first year after the event and a substantial proportion of the survivors are left with serious neurological deficits. About 25 to 30 % of patients deteriorate within first 24 hours in hospital because of hematoma growth [12]. Thus, there is an urgent need for a simple diagnostic test which can help in the hospital management of ICH patients.

Several prospective studies have reported that increased levels of acute inflammatory markers, such as C-reactive protein (CRP), and white blood cell (WBC) count are associated with increased risk of death or disability. Greater changes in leukocyte count over the first 72 hours after admission predicted both short term and long term worse and functional outcomes after ICH [1]. Blood glucose (BG) represents a novel prognostic marker in acute ICH, playing a major role in the pathogenesis of the acute inflammatory response in ICH patients. The prognostic role of these inflammatory markers after ICH is less clear. Early prediction of outcome in patients with ICH is important and biomarkers might allow the individualization of care by stratifying risk of reperfusion haemorrhage, predicting relative volume of penumbral tissue, and providing additional prognostic information. In this study we propose to investigate the role of CRP level, serum glial fibrillary acidic protein, troponin, change in leukocyte count, S100B, copeptin levels as independent predictors of the neurological outcome in patients with primary intracerebral haemorrhage.

### Copeptin

Copeptin has emerged as a new diagnostic and prognostic biomarker in various diseases, but its prognostic value in ICH is still unknown. Its level is high in patients with ICH. One study suggested that copeptin levels were higher in patients who died in 30 days than in 30 days survivors. Its levels were also higher in patients with an unfavourable clinical outcome at 90 days in ICH [26]. Increase in level of plasma copeptin is an independent prognostic marker of 1-year mortality, 1-year unfavorable outcome and early neurological deterioration [25] and associated with mortality and outcome in patients with ICH [26]. Copeptin is a new prognostic marker in patients with ICH, wrote Zweifel and colleagues, University Hospital, Department of Neurosurgery and also suggested that “if this finding can be confirmed in larger studies, it might serve as an additional valuable tool for risk stratification and decision making in ICH patients.” In this study we will assess the level of copeptin and identify its relationship with the prediction of outcome in ICH patients.

### Troponin

Cardiac troponin level is being used as a test of choice for the detection of myocardial injury. One study suggested that in surgically treated ICH, elevated cardiac troponin levels are predictor of mortality and should be considered in managing the decisions of ICH [13]. Higher level of troponin on admission is a significant risk factor for in-hospital mortality in haemorrhagic patients [3]. Elevated level of cardiac troponin has been associated with adverse prognosis in patients with acute neurological diseases. Only few studies have been conducted to know the relation between cTnT and prognosis of ICH, but results are not conclusive [17,21]. In this study, we will identify the relationship between cTnT and outcome in patients with spontaneous ICH.

### S100B

S100B is a member of calcium-mediated low molecular weight glial protein (approximately 10 kDa). Various combinations of subunits (α and β) make up the S100 protein family, which diverge into the hetero- and homodimer forms of (α and β) subunits. S100B is comprised of the (α-α, α- β, β-β). The highly specific form is α- β, β-β forms, to nervous tissue, and is found in abundance in the cerebral astroglial compartment, peripheral Schwann cells, and extraneuronally in melanocytes, adipocytes, and chondrocytes [9]. The concentration of S100B is 40-fold higher in CSF than in serum. Serum S100B levels after injury accurately predicts neurological function at discharge after supratentorial ICH in the first 24 h [18]. We hypothesize that S100B could be useful as a biomarker for ICH patients.

### Leukocyte count

After the spontaneous ICH, increase in peripheral leukocytes is an important marker of response of the immune system and causes the activation of the inflammatory cascade [2]. This is the most routinely used biomarker to know the amount of inflammation mounted with 72 hours after the intracerebral haemorrhage. Measurement of leukocyte count after the spontaneous intracerebral haemorrhage may accurately reflect the extent of neuroinflammation. It independently predicts poor functional outcomes in terms of discharge disposition [1]. We hypothesize that change in leukocyte count could be useful as a biomarker for ICH patients.

### C - reactive protein

C-reactive protein (CRP) is an inflammatory protein and its level rises in response to inflammation in an acute phase of inflammatory reactions. It binds with phosphocholine expressed on the surface of dead or dying cells in order to activate the complement system. It also has a role in complex modulatory functions. It may directly participate in enhancing inflammation in cerebral vessels and brain injury through activation of complement cascade, initiation of leukocyte chemotaxis and expression of adhesion molecules through a positive feedback mechanism [15]. It also induces apoptosis through a caspase-dependent mechanism [4]. Data are limited regarding the role of CRP in the pathophysiological mechanisms and predictive outcome after sICH. Higher levels of CRP are significantly associated with 30-day death after sICH [7] and an independent predictor of poor outcome in intracerebral haemorrhage [8]. We hypothesize that it could be an important predictor of outcome in ICH patients.

### Glial fibrillary acidic protein (GFAP)

Glial fibrillary acidic protein (GFAP) is a member of cytoskeleton protein family. It is expressed by numerous cell types of the central nervous system including ependymal cells and astrocyte cells. It helps to maintain astrocyte mechanical strength, as well as the shape of the cells. It is evident that GAP is considered to be important sensitive and specific marker for the rapid astrocyte response to injury and disease. It is also involved in promotion of normal blood brain barrier. One study indicated that serum GFAP may function as a reliable biomarker for intracerebral haemorrhage in acute stroke. Increased GFAP levels are associated with the blood brain barrier injury [20]. GFAP may be useful as a surrogate marker and may be helpful for management of hemorrhagic stroke [12]. We hypothesize that GFAP is immediately detectable in serum in acute phase of ICH and could be useful as a biomarker for ICH patients.

A prediction model based on single or multiple biomarkers may help in clinical management, if it can predict haematoma growth or long-term outcome. At present, there is no single biomarker or panel of biomarkers that has the level of accuracy or precision to be useful in clinical practices. A combination of clinical, neuroimaging and biomarkers may be able to achieve this objective. The main objectives of the present study are (i) to determine whether any of the biomarkers S100B, Copeptin, CRP, Leukocyte count, Troponin, Glial fibrillary acidic protein are independent predictors of the neurological outcome in patients with primary intracerebral haemorrhage, and (ii) whether any of them (singly or in combination) improve the predictive accuracy of clinically important outcomes.

## Methods/design

### Ethical considerations

Study Protocol has been approved from Institute Ethics Committee.

### Design of study

Prospective cohort study.

### Inclusion and Exclusion criteria

Patients will be eligible if they meet all the inclusion criteria and none of the exclusion criteria.

#### Inclusion criteria

Patients will be judged eligible if they have all of the following: (1) Sudden onset of focal neurological deficit or impairment of consciousness; (2) Age greater than 18 years; (3) CT scan showing parenchymal haematoma in brain; (4) Admission within 72 hours after the onset of the qualifying event; (5) Accessibility for follow-up by telephone (landline or mobile).

#### Exclusion criteria

Patients meeting the inclusion criteria will be considered ineligible for the study if they have any one of the following: (1) Suspicion or documented history of a bleeding disorder; (2) History of recent head trauma; (3) History of ingestion of anticoagulant drugs within seven days of onset of stroke; (4) A documented A-V malformation; aneurysm or cerebral neoplasm as the underlying cause of primary supratentorial intracerebral haemorrhage; (5) Pre-morbid organ failure or disability leading to dependence on others for activities of daily living; (6) Unwillingness to provide written informed consent (by self or next of kin); (7) Hemorrhagic transformation of cerebral infarct; (8) Concurrent major renal or hepatic disease; (9) Pregnancy.

#### Centre’s eligibility criteria

(1) Be a Department of Scientific and Industrial Research (DSIR) certified or a medical college or government hospital; (2) Admitting at least 5 patients with stroke per month; (3) Have access to CT scan; computer and internet; (4) Have access to a laptop and refrigerator with −80 °C.

### Consent and Recruitment

Witnessed written informed consent will be requested from all eligible patients or their next of kin (in case of patients with aphasia or impaired consciousness). Literate subjects or their next of kin will be requested to give their written consent by one of the physician investigators with a nurse witnessing the consent. Subjects or their next of kin who are not literate will have the contents of the consent form read out and explained by the investigator and their left thumb impression will be taken according to the current practice with a nurse as a witness. All questions from the subjects or their next of kin will be answered by the investigator.

Potentially eligible patients will be recruited from the emergency services and ward of the participating centre. All potentially eligible patients will undergo emergency head CT for diagnosis of primary intracerebral haemorrhage. All patients with primary intracerebral haemorrhage will be admitted and their eligibility will be assessed by the investigators. Patients suspected to have aneurysm or arteriovenous malformation will be excluded unless digital subtraction angiography. A logbook of all patients screened, those eligible will be maintained. This log will be used to estimate the proportion of all potentially eligible patients who enter the study and will be used to assess the generalizability of study results.

### Baseline assessment

Standardized forms will be used to record patient history, general and neurological examination. Clinical, laboratory and radiological findings to be recorded at baseline will include:

#### A. Clinical assessment

Age, Sex, History of hypertension, diabetes, smoking and family history of stroke or coronary artery disease, Treatment history for hypertension and diabetes, if known to have one or both of these, Time of onset of the stroke, Blood pressure at the time of recruitment, Glasgow Coma Scale (GCS) score at the time of recruitment, Barthel index (BI) score at the time of recruitment, National Institute of Health Stroke Scale (NIHSS) and ICH Score.

#### B. Radiological assessment

Site and volume of haematoma and any midline shift as determined on CT scan, Intraventricular extension of the haematoma as determined by CT scan, CT scans will be uploaded on the day of recruitment using software to be installed by AIIMS neuro-radiology department. The scans will be viewed for quality on the same day, and if not satisfactory, repeat CT will be done within hours.

#### C. Biomarker Assay and Sample transportation

After the written informed consent of patient/relatives venous blood sample (10 ml) will be collected in a specific serum vacutainer and EDTA coated vial. For serum collection it will be left standing at room temperature for 30 min until clotted. It will then undergo centrifugation at 3000 rpm for 15 min, after which the serum will be separated into two equal aliquots in serum containing vials. Both aliquots will be stored at −80 °C. EDTA tubes will be centrifuged for plasma separation and stored at −80 °C until analysis. It will be packed within a month at participating centres and transferred to Principal Centre (AIIMS) for the biomarker analysis. Prior to shipment the serum samples will be removed from the −80 °C refrigerator and will be placed inside one of two identical reusable thermal insulated pouches. A single frozen ice pack, gel pack and dry ice will be placed inside one of the two insulated pouches and sealed. Samples will be transferred by using standard courier service to the AIIMS by priority overnight delivery. Sample will be used to assay the biomarker levels using available methods as mentioned in Table 1.

**Table 1:** List of Biomarkers to be assayed

S.No	Name of Biomarker	Source	Method of Assessment
1	S100B	Plasma	ELISA [6]
2	Copeptin	Plasma	ELISA [10]
3	CRP	Serum	Immunoturbidimetric assay [7]
4	Glial fibrillary acidic protein	Serum	ELISA [19]
5	Leukocyte count	Blood	Automated cell counters [5]
6	Troponin	Serum	ELISA [21]

### Sample Size

As this study involves multiple variables (11 clinical, 03 neuroimaging and 06 biomarkers), we plan to use prognostic modelling. According to Harrell et al. [16] a stable and replicable model will require 20 events per variable. As the outcome events in ICH over six months has a frequency of approximate 50 % [24], and 23 variables are planned to be examined, we need 920 patients with complete follow up. Adjusting this for 10 % loss to follow up, yields a sample size of 1020.

### Patient management

All subjects will receive standard medical therapy consisting of maintenance of adequate airway, fluids and electrolyte balance, and good pulmonary and cardiac function. Nasogastric feeding will be instituted in unconscious patients. Patients who experience a seizure at any time since onset of the stroke will be given a loading dose of an anticonvulsant intravenously (usually phenytoin 15–20 mg/kg body weight i.v. push) followed by a maintenance dose (usually phenytoin 5 mg/kg divided over a period of three time a day i.v. push) until recovery of consciousness when oral administration of the same dose will be continued for six months. The dose and the drug will be adjusted to minimize the side-effects. Severe hypertension (defined as systolic BP more than 200 mg Hg and diastolic BP more than 120 mm Hg) will be treated with diuretics and/or ACE inhibitors so as to keep systolic BP between 160–200 mm Hg and diastolic BP between 100–110 mm Hg. Corticosteroids, glycerol or antithrombotics will not be used. Hyperventilation will be used to control intracranial pressure especially when signs of brain herniation develop. The medical management of all patients will be carried out by a team of neurologists and neuroanaesthetists in the intensive care unit settings according to National guidelines [22]. Patients meeting the criteria according to the National guideline will be operated. All interventions administered to the patients will be recorded and documented for trial purposes.

### In-hospital follow-up

All subjects will be assessed daily by the neurologist investigators with the help of neurology residents. CT scan will be repeated between 24 to 48 hours to detect haematoma growth. Serum chemistry and blood gases will be monitored daily for unconscious patients. Other investigations will be done when clinically indicated. A ward physiotherapist will see each subject twice a week and implement an appropriate physiotherapy plan. Nurses will provide nursing care to all subjects regularly. All subjects will be observed in the hospital for a minimum period of 30 days post-inclusion with the exception of those who are conscious and desire to leave the hospital early.

### Outpatient follow up

Central telephonic follow-up will be done at three months, six months and then on annual basis till the end of the study. This data will be used to study the frequency and determination of recurrent stroke in this population on long term basis (such data is not yet available from India).

### Strategies to minimize losses to follow-up

All the patients will be followed centrally by telephone. For this, two to five telephone numbers (both mobile and landline) will be noted from the patients or his/her relatives at the time of recruitment. A research worker trained and certified in modified Rankin scale (mRS) will administer the scale to all patients (or his representative) from AIIMS, blinded to the baseline clinical or neuroimaging scores or level of biomarkers.

On failure to contact, the worker will attempt to obtain a new contact number from the relatives or other contacts and administer the scale.

### Outcomes and their measurement

The primary endpoint will be death at 30 days. Glasgow Outcome Scale (GOS) will be used to determine the functional recovery at 30 days. Good clinical outcome will be considered if GOS score would be 4 and 5 and poor outcome if score would be 1–3.

A research worker trained and certified in modified Rankin scale will administer the scale at three months and six months to all patients blinded to the baseline clinical or neuroimaging scores or result of biomarkers.

Barthel index at three months and six months post-inclusion will be measured centrally by blinded and trained research worker by telephone in a binary scale as ‘independent’ or ‘dead/dependent’. ‘Independent’ is defined as the Barthel index score of 60 or more. Surviving subjects who do not achieve this are classified as ‘dependent’. Favorable clinical outcome: defined as any of the following:

- A score of 0 to 3 on mRS at 180 days
- A Barthel Index score of 60 to 100 at 180 days

### Statistical Method

After data clearing and rectification bivariate analysis will be done to determine association between the predictor variable and outcome. Continuous variables will be described by mean and SD and categorical variables by percentage. Association between biomarkers and radiological score would be done by linear regression methods. For examining independence of the association, logistic (for dichotomous dependent variable) or cox regression (for time to event variable) will be used. For determining accuracy and precision, prognostic models will be developed using standard methods and prediction rules will be framed.

## Discussion

This study will aim at predicting the poor short term or long term prognosis with the use of clinical neuroimaging and biomarkers such as CRP, GFAP, troponin, change in leukocyte count, S100B and copeptin in order to help clinicians to stratify patients for early referral/intervention.
